# Application of stem cells and exosomes in the treatment of intracerebral hemorrhage: an update

**DOI:** 10.1186/s13287-022-02965-2

**Published:** 2022-06-28

**Authors:** Jian-feng Zhou, Yu Xiong, Xiaodong Kang, Zhigang Pan, Qiangbin Zhu, Roland Goldbrunner, Lampis Stavrinou, Shu Lin, Weipeng Hu, Feng Zheng, Pantelis Stavrinou

**Affiliations:** 1 Department of Neurosurgery, The Second Affiliated Hospital, Fujian Medical University, No. 34 North Zhongshan Road, Quanzhou, 362000 Fujian China; 2 Department of Neurosurgery, Hui’an County Hospital of Fujian Province, Quanzhou, Fujian China; 3 Department of Neurosurgery, Faculty of Medicine and University Hospital, Center for Neurosurgery, University of Cologne, Cologne, Germany; 4 2nd Department of Neurosurgery, Athens Medical School, “Attikon” University Hospital, National and Kapodistrian University, Athens, Greece; 5 Centre of Neurological and Metabolic Research, The Second Affiliated Hospital of Fujian Medical University, No. 34 North Zhongshan Road, Quanzhou, 362000 Fujian China; 6 Diabetes and Metabolism Division, Garvan Institute of Medical Research, 384 Victoria Street, Darlinghurst, Sydney, NSW 2010 Australia; 7 Neurosurgery, Metropolitan Hospital, Athens, Greece

**Keywords:** Intracerebral hemorrhage, Exosomes, Neuroprotection, Stem cells, Mesenchymal stem cells

## Abstract

Non-traumatic intracerebral hemorrhage is a highly destructive intracranial disease with high mortality and morbidity rates. The main risk factors for cerebral hemorrhage include hypertension, amyloidosis, vasculitis, drug abuse, coagulation dysfunction, and genetic factors. Clinically, surviving patients with intracerebral hemorrhage exhibit different degrees of neurological deficits after discharge. In recent years, with the development of regenerative medicine, an increasing number of researchers have begun to pay attention to stem cell and exosome therapy as a new method for the treatment of intracerebral hemorrhage, owing to their intrinsic potential in neuroprotection and neurorestoration. Many animal studies have shown that stem cells can directly or indirectly participate in the treatment of intracerebral hemorrhage through regeneration, differentiation, or secretion. However, considering the uncertainty of its safety and efficacy, clinical studies are still lacking. This article reviews the treatment of intracerebral hemorrhage using stem cells and exosomes from both preclinical and clinical studies and summarizes the possible mechanisms of stem cell therapy. This review aims to provide a reference for future research and new strategies for clinical treatment.

## Introduction

Non-traumatic intracerebral hemorrhage (ICH), which accounts for almost 15% of strokes, is a highly destructive intracranial disease associated with high mortality and morbidity1, half the deaths occur in the first two days post-stroke, and the mortality rate is as high as 40% within the 1st month after the onset of a stroke2. ICH can significantly reduce patients’ quality of life and place a huge economic burden on the family and society, as most survivors experience severe neurological deficits. The main risk factors associated with ICH include hypertension, amyloidosis, vasculitis, drug abuse, coagulation dysfunction, and genetic factors 3–6. Genetically, gene polymorphisms, such as endogenous tissue inhibitors of metalloproteinases; tumor necrosis factor-α (TNF-α); interleukin (IL), angiotensin-converting enzyme, coagulation factor XIII, and plasminogen activator inhibitor-1 genes; and point mutations, including Ras homolog family member A (RhoA), apoproteins H (apoH), and platelet-activating factor acetylhydrolase, may be associated with ICH [7–14].

Brain injury after cerebral hemorrhage can be primary or secondary. In primary brain injury, blood vessels rupture to form a hematoma, causing direct mechanical damage owing to local compression. Hematoma expansion further compresses the surrounding normal brain tissue, resulting in loss of nerve function [15]. Hematomas not only cause mechanical compression but also induce toxicity, inflammation, oxidative stress, and apoptosis-of-hematoma metabolites, which can further damage the nervous system and cause secondary brain injury [16–18]. Surgical removal of a compressing hematoma, when indicated, may relieve intracranial pressure, prevent brain herniation, improve cerebral blood perfusion, remove toxic substances, reduce cerebral edema severity, and improve survival [19]. Hematoma evacuation techniques range from traditional craniotomy to minimally invasive techniques, such as neuroendoscopic hematoma removal and stereotactic hematoma aspiration. However, these treatments have a moderate effect on outcomes, and despite surgical evacuation or optimal medical treatment, the prognosis of ICH remains poor [20, 21]. Experimental results have been promising but have not yet been translated into therapeutic benefits. Recently, with the growing popularity of regenerative medicine, research has focused on stem cell and/or exosome therapy for ICH. Stem cells have been widely used in the study of ICH owing to their strong reproductive ability, low differentiation degree, multi-lineage differentiation potential, anti-inflammatory properties, low immunogenicity, immunomodulatory ability, and secretory ability. Exosomes derived from stem cells are also sought after, because they possess some of the characteristics and transport capabilities of primary cells [22].

In addition, stem cells and exosomes can be harvested from a wide range of sources, including embryos, bone marrow, umbilical cord, adipose tissue, nerve tissue, and induced somatic cells [23–26]. Numerous animal studies have shown that stem cells can directly or indirectly participate in ICH treatment through regeneration, differentiation, or secretion. However, considering the uncertainty of its safety and efficacy, clinical studies are still lacking. This article reviews preclinical and clinical studies of ICH treatment using stem cells and exosomes and summarizes the possible mechanisms of stem cell therapy. Simultaneously, it provides a reference for future research and “point-to-point therapy.”

## Stem cells sources for ICH treatment

Stem cells have unlimited reproductive capacity and can differentiate into cells with specific functions. Depending on their differentiation potential, they can be divided into totipotent, pluripotent, and multipotent stem cells. In a broad sense, totipotent stem cells can produce all extraembryonic tissues and can differentiate into any type of cell in the body, such as blastomeres. Pluripotent stem cells, such as embryonic stem cells (ESCs) and induced pluripotent stem cells (iPSCs), can only differentiate into certain cells in the three germ layers. Multipotent stem cells, including mesenchymal stem cells (MSCs) and neural stem cells (NSCs), can produce all cell types within a particular lineage [27–29].

**Fig. 1:**
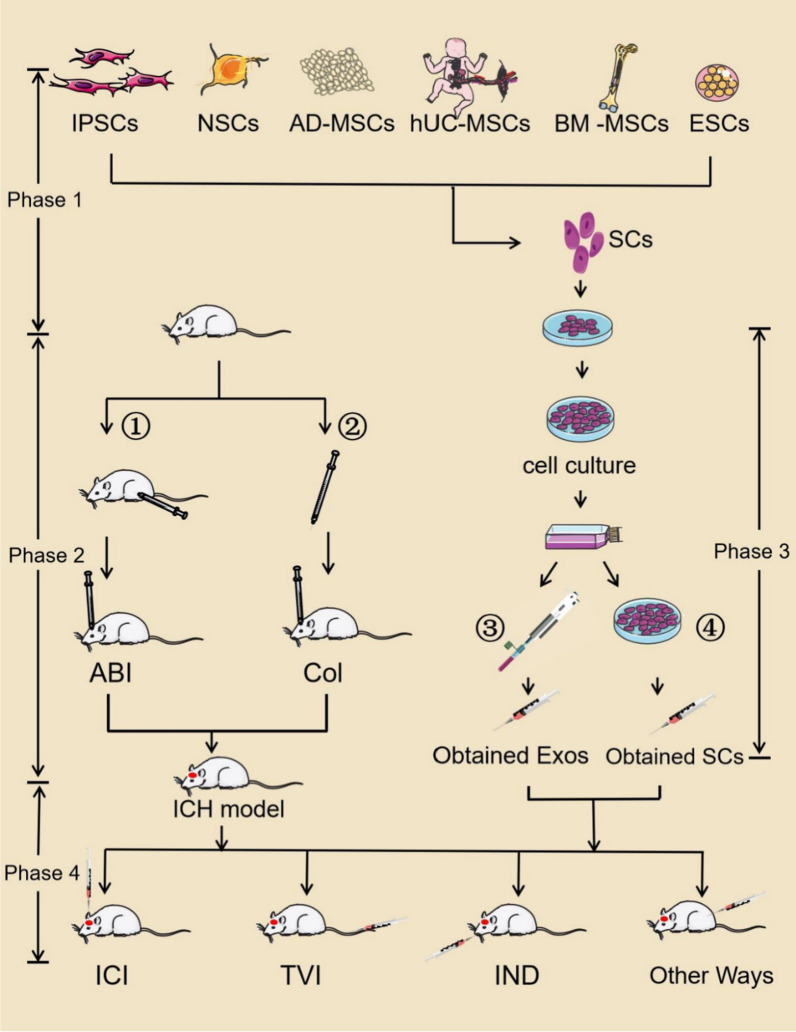
Stem cells and stem cell-derived exosomes are used to treat ICH mice. Phase 1: Cell extraction: stem cell extraction from various sources followed by cultivation and extraction of stem cells or exosomes as required (③, ④). Phase 2: ICH animal model establishment: cerebral hemorrhage model through autologous blood or collagenase stereotactic injection (①, ②). Phase 3: cell culture and extraction of stem cells or exosomes. Phase 4: Treat ICH animals with different administration methods. IPSCs: induced pluripotent stem cells; NSCs: neural stem cells; AD-MSCs: adipose-derived mesenchymal stem cells; hUC-MSCs: human umbilical cord mesenchymal stem cells; BM-MSCs: bone marrow mesenchymal stem cells; ESCs: embryonic stem cells; ICH: intracerebral hemorrhage; ABI: autologous blood injection; Col: collagenase; ICI: intracerebral injection; TVI: tail vein injection; IND: intranasal deliver; Exos: exosomes; and SCs: stem cells

In order to simulate a human ICH, two mouse models of cerebral hemorrhage have been mainly used: the collagenase model, where injection of a bacterial enzyme digests the collagen present in the basal lamina of the blood vessels to produce spontaneous brain hemorrhage [30], and the blood infusion model, where a fixed amount of blood (autologous or donor) is injected into the brain parenchyma to mimic a primary accumulation, similar to human ICH [31]. In the former, the bleeding process mimics the continuous process in patients with ICH, and mice show more typical and obvious neurological dysfunction. Owing to its rapid emergence, the latter model can better mimic brain tissue damage from hematoma pressure and blood outside the blood vessels. The blood infusion model best mimics the nerve recovery process of patients with ICH [32, 33]. Therefore, different model establishment methods can be chosen for the intended research purpose. After establishing a good ICH animal model, researchers can inject stem cells or exosomes through a variety of routes for treatment, as shown in Fig. 1

### Pluripotent stem cells

Pluripotent stem cells can be found in blastocysts and early embryonic developmental stages and can differentiate into multiple cell lineages [34]. These stem cells include mesenchymal stem cells (MSCs) and iPSCs.

#### Embryonic stem cells

In 1981, pluripotent ESCs were first extracted from in vitro mouse blastocysts [23]. These cells are highly undifferentiated and can self-renew, reproduce indefinitely, mature, and differentiate into any cell, including nerve cells [35, 36]. In 1995, ESCs were extracted from primates [37] and 3 years later from human cell lines. ESCs exist only during the early stages of embryonic development [38]. Because ESCs are capable of multidirectional differentiation, they have been used for in vitro and in vivo regeneration studies. Nonaka et al. used collagenase to establish an ICH rat model and utilized all-trans retinoic acid (ATRA) to induce nestin-positive neural stem cells in vitro. After 7 days of model establishment, ATRA-treated ESCs were infused into the healthy hemispheres. Ultimately, researchers have found neurons and astrocytes originating from ESCs in the area around the hematoma, highlighting the therapeutic potential of ESCs [39]. Owing to its low migratory ability, cell therapy for ICH remains a challenge. To solve this problem, Kang et al. selected safe and efficient ferromagnetic iron oxide nanocubes to label spherical neural masses derived from human embryonic stem cells and used magnetically embedded helmets for targeted delivery under an external magnetic field. In the experimental group, the hematoma volume and edema area were reduced, the infiltration capacity of inflammatory cells was low, and the inflammation cascade was weakened [40].

To date, we have identified very few publications regarding ESCs in cerebral hemorrhage, with even fewer human clinical trials. This is primarily due to two reasons. Although human ESCs (hESCs) can theoretically differentiate into more than 200 types of human cells, in reality, the majority of cell types are difficult to generate, and only 10 types of cells are truly functionally equivalent to normal human cells. Moreover, ESCs are difficult to separate and acquire. Moreover, ESCs may have oncogenic potential, raising serious concerns regarding their safety in clinical studies [41].

#### Induced pluripotent stem cells

In 2006, Takahashi et al. transported four exogenous genes (OCT4, KLF4, SOX2, and c-MYC) into somatic cells and successfully induced iPSCs, a new pluripotent stem cell line [25]. These newborn stem cells express marker genes, growth characteristics, and embryonic stem cell morphology. Subsequently, the distinctive features of these cells were revealed: iPSCs can replace injured or diseased tissue, resist inflammation, secrete biologically active substances, and exhibit low immunogenicity [42]. Eight years later, iPSCs were first implanted in patients with ophthalmopathy [43]. As pluripotent stem cells, iPSCs can differentiate into nerve cells and neuroepithelium-like/neuroepithelioid stem cells with secretory function. Qin et al. successfully transfected all the above-mentioned transcription factors into the fibroblasts of patients with ICH using viral vectors and eventually obtained iPSCs. In vitro, these cells were cultured in a fibroblast medium to induce differentiation of iPSCs into neural cells and neuroepithelial-like stem cells (NESCs). After injecting the differentiated cells into rats, nerve function was significantly improved, and NESCs appeared in the lesion area. Differentiated nerve cells can play a role in replacing damaged cells [44]. In addition to the site of differentiation and injury, subsequent experiments by Qin et al. found that iPSCs secreted brain-derived neurotrophic factor (BDNF) and vascular endothelial growth factor (VEGF) cytokines and inflammation-related factors (IL-1β, IL-6, TNF-α, and IL-10) during treatment [45, 46].

Although iPSCs have been available for less than 20 years, much research has been conducted [25]. However, few clinical and animal studies on iPSCs have been conducted. This seems related to the inefficiency of reprogramming, which represents a persistent problem in both laboratory and clinical studies. More importantly, in many experimental studies, the uncontrolled reproduction of iPSCs ultimately led to the formation of teratomas [47–50].

### Multipotent stem cells

As an integral part of the stem cell family, multipotent cells differentiate into all cell types within a particular lineage. Mesenchymal stem cells and neural stem cells, as their representatives, are often used in preclinical and clinical studies [29].

#### Mesenchymal stem cells

In addition to the general differentiation and proliferation potential, MSCs proposed by the International Society for Cell Therapy must meet three minimum requirements. First, MSCs must adhere to plastic when maintained in standard culture conditions. Secondly, MSCs-surface markers must positively express CD73, CD90, and CD105 and negatively express CD11b or CD14, CD19 or CD79a, CD34, and CD45. Third, in vitro, they should be able to differentiate into osteoblasts, adipocytes, and chondroblasts [51] (Table 1). MSCs can be divided into bone marrow mesenchymal stem cells (BM-MSCs), human umbilical cord mesenchymal stem cells (hUC-MSCs), and adipose-derived mesenchymal stem cells (AD-MSCs) [26].

**Table 1:** Minimum standards for mesenchymal stem cell identification

1. Ability to adhere to plastic under standard culture conditions [51];		
2. Under standard in vitro differentiation conditions, cells can differentiate into adipocytes, osteoblasts and chondroblasts;		
3. Cell surface markers	Positive (≥ 95%) CD73 CD90 CD105	Negative (≤ 2%) CD34 CD45 CD14 or CD11b CD19 or CD79α HLA-DR

##### Bone marrow mesenchymal stem cells

BM-MSCs are stem cells derived from bone marrow. They possess multiple functions such as secretion, self-renewal, and differentiation [52, 53]. These cells can differentiate into chondroblasts, adipocytes, osteoblasts, and neural cells. BM-MSCs have the advantages of a simple extraction process, rapid expansion in vitro, low antigenicity, sufficient donor sources [54, 55], and the ability to pass through the blood–brain barrier (BBB) [56]. Hence, BM-MSCs have become a popular source of stem cells.

Researchers have found that bone marrow mesenchymal stem cells can not only differentiate into target cells and promote endogenous neurogenesis but may also reverse the ICH-induced destruction of the blood–brain barrier [57, 58]. In the ICH mouse model, exogenous BM-MSCs were injected into the brain parenchyma of ICH mice. The implanted cells differentiated into neurons and astrocytes, which promoted endogenous neurogenesis [59]. Meanwhile, intranasal injection of hypoxia-pretreated bone marrow mesenchymal stem cells into ICH mice promoted endogenous neurogenesis, induced the expression of neurotrophic factors, and improved functional defects [60] (Table 2). Additionally, BM-MSCs can improve the prognosis of ICH by genetic modification. Taking advantage of adenoviruses, BM-MSCs were eventually transfected into genetically engineered cells. Glial cell-derived neurotrophic factor (GDNF)-transduced rat bone marrow MCSs were implanted into ICH rats. Compared with BM-MSCs, modified cells can preferentially transform into astrocytes, which improves the outcome of sensory, motor, balance, and reflex tests, reduces lesions, and inhibits apoptosis [71]. Liang reported that the limb function of rats with cerebral hemorrhage recovered well after injection of BM-MSCs. The proportion of brain reorganization increased post-treatment, but the hematoma volume size of the experimental group was not significantly different after treatment, inconsistent with previous studies. Although BM-MSCs may not be able to prevent ICH-induced pontine atrophy, they can enhance corticospinal tract nerve regeneration and effectively improve nerve function recovery [69].

**Table 2:** Preclinical studies of stem cell therapy for ICH

References	Drug delivery route	Number of cells/exosomes	Type of ICH model Rat	Sample Size	Treatment Day after ICH	Earliest effective time (post ICH)	Behavioral recovery
Nonaka et al. [61]	Intraventricular	1 × 10^5^ ESCs	Rat Col VII	Sham 5 Treatment 10	7 d	NA	NA
Chen et al. [62]	Intraventricular	2–4 × 10^5^ AD-MSCs	Rat Col IV	Control 40 Treatment 40	2 d	3 d	Zea Longa 5-grade scale↑
Cui et al. [63]	Intravenous	5 × 10^6^ MSCs	Rat ABI	Control 15 Treatment 15	1 h and 24 h	3 d	NSS↓
Wang et al. [38]	Intravenous	1 × 10^6^ MSCs	Rat ABI	Sham 12 Control 24 Treatment 24	NA	14 d	mNSS↓ MLPT scores↓
Chen et al. [39]	Intravenous	5 × 10^6^ MSCs	Rat Col IV	NA	2 h	3 d	mNSS↓
Wang et al. [42]	Intravenous	1 × 10^6^ MSCs	Rat Col VII	Control 6 treatment6	1 h	7 d	mNSS↓

References	Drug delivery route	Number of cells/exosomes	Type of ICH model Rat	Sample Size	Treatment Day after ICH	Earliest effective time (post ICH)	Behavioral recovery
Seyfried et al. [52]	Intravenous	0.5–1 × 10^6^ MSCs	Rat ABI	Control 9 Treatment 18	24 h	7 d	mNSS↓ corner-turn test↑
Nan et al. [57]	Intravenous	2.4–3.2 × 10^6^ MSCs	Rat Col VII	NA	24 h	7 d	limb-placement tests↑ stepping tests↑ body-swing tests↑
Yang et al. [64]	Intravenous	1 × 10^6^ AD-MSCs	Rat Col VII	Control 6 Treatment 9	24 h	7 d	mNSS↓
Kim et al. [65]	Intravenous	3 × 10^6^ AD-MSCs	Rat Col VII	Control 16 Treatment 16	24 h	28 d	MLPT scores↓
Jeong et al. [66]	Intravenous	5 × 10^6^ NSCs	Rat Col V	Control 13 Treatment 12	24 h	14 d	rotarod test ↑ MLPT scores ↓
Vaquero et al. [67]	Intracerebral	5 × 10^6^ MSCs	Rat Col V	Control 20 Treatment 20	2 m	4 m	rotarod tests↑ VTB tests↑
Bao et al. [68]	Intracerebral	2 × 10^5^ MSCs	Rat Col VII	Control 67 Treatment 65	24 h	3 d	mNSS↓

References	Drug delivery route	Number of cells/exosomes	Type of ICH model Rat	Sample Size	Treatment day after ICH	Earliest effective time (post ICH)	Behavioral recovery
Liang et al. [69]	Intracerebral	1 × 10^6^ MSCs	Rat Col IV	Sham 8 Control 16 Treatment 16	24 h	7 d	MLPT scores ↓ vibrissae-elicited forelimb-placing test↑
Otero et al. [70]	Intracerebral	2 × 10^6^ MSCs	Rat Col IV	Control 48 Treatment 48	2 h	NA	NA
Yang et al. [71]	Intracerebral	5 × 10^5^ MSCs	Rat Col I	Control 10 Treatment20	3 d	7 d	mNSS↓
Otero et al. [72]	Intracerebral	2 × 10^6^ MSCs	Rat Col IV	Control 10 Treatment10	3 d	3 w	mNSS↓ Rotarod test↑
Otero et al. [73]	Intracerebral	5 × 10^6^ MSCs	Rat Col IV	Control 10 Treatment 10	2 m	3 m	mNSS↓ Rotarod tests↑ VTB tests↑ locomotor activity↑
Feng et al. [74]	Intracerebral	1–5 × 10^6^ MSCs	Monkey ABI	Control 8 Treatment16	1 w or 4 w	2 w or 5 w	Neurologic deficit scores↓

References	Drug delivery route	Number of cells/exosomes	Type of ICH Model Rat	Sample Size	Treatment day after ICH	Earliest effective time (post ICH)	Behavioral recovery
Nagai et al. [75]	Intracerebral	2 × 10^5^ MSCs	Rat Col VII	Control 4 Treatment 14	7 d	8 d	Rotarod test↑
Seyfried et al. [76]	Intracerebral	3, 5 and 8 × 10^6^ MSCs	Rat ABI	Control 27 Treatment 27	24 h	7 d	mNSS↓ corner-turn test↑
Zhang et al. [77]	Intracerebral	1 × 10^5^ MSCs	Rat Col IV	Control 60 Treatment 60	6 h	24 h	mNSS↓
Gao et al. [78]	Intracerebral	5 × 10^5^ NSCs	Rat Col IV	Sham 20 Control 48 Treatment 26	3 h	3 d	mNSS↓
Wakai et al. [79]	Intracerebral	1 × 10^5^ NSCs	Mouse ABI	NA	3 d	21 d	Cylinder tests↑ corner-turn tests↑
Wang et al. [80]	Intracerebral	1 × 10^6^ NSCs	Rat Col VII	Control 26 Treatment 13	3 d	24 d	MLPT scores↓
Lee et al. [81]	Intracerebral	2 × 10^5^ NSCs	Mouse Col IV	Control 28 Treatment 61	7 d	8 d	Rotarod test↑ MLPT scores↓

References	Drug delivery route	Number of cells/exosomes	Type of ICH Model Rat	Sample Size	Treatment day after ICH	Earliest effective time (post ICH)	Behavioral recovery
Lee et al. [82]	Intracerebral	2 × 10^5^ NSCs	Mouse Col IV	Control 20 Treatment 18	7 d	5 w	Rotarod test↑ MLPT scores↓
Lee et al. [83]	Intracerebral	2 × 10^5^ NSCs	Mouse Col IV	Control 18 Treatment 39	7 d	8 d	Rotarod test↑ MLPT scores↓
Lee et al. [84]	Intracerebral	2 × 10^5^ NSCs	Mouse Col VII	Control 30 Treatment 31	7 d	21 d	Rotarod test↑ MLPT scores↓
Lee et al. [85]	Intracerebral	2 × 10^5^ NSCs	Mouse Col IV	Control 20 Treatment 50	7 d	8 d	Rotarod test↑ MLPT scores↓
Qin et al. [46]	Intracerebral	1 × 10^6^ iPSCs	Rat Col VII	Sham 30 Control 63 Treatment 59	6 h	14 d	MLPT scores↓
Qin et al. [44]	Intracerebral	2 × 10^6^ iPSCs	Rat Col VII	NA	24 h	14 d	mNSS↓ MLPT scores↓
Qin et al. [45]	Intracerebral	1 × 106 iPSCs	Rat Col VII	Sham 12 Control 24 Treatment 12	24 h	14 d	mNSS↓ MLPT scores↓

References	Drug delivery route	Number of cells/exosomes	Type of ICH model Rat	Sample size	Treatment day after ICH	Earliest effective time (post ICH)	Behavioral recovery
Seyfried et al. [86]	Intra-arterial	1 × 10^6^ MSCs	Rat ABI	Control 18 Treatment 18	24 h	7 d	mNSS↓ corner-turn test↑
Li et al. [87]	Intra-arterial	4 × 10^6^ NSCs	Rat Col VII	Control 8 Treatment 40	2, 7, 14, 21 or 28 d	28 d	Rotarod tests↑ beam-walking tests↑ limb-placing tests↑ spontaneous cycling tests↑
Zhang et al. [88]	Intra-arterial intravenous intraventricular	2 × 10^6^ MSCs	Rat Col VII	Blank 5 Sham 20 Control 20 Treatment 60	1, 3, 5 and 7 d	24 h	Beam-walking test ↑ (intravenous group →)
Xie et al. [89]	Intracerebral, intravenous	2 × 10^5^(IC) 2 × 10^6^(IV) MSCs	Rat Col VII	Sham 10 Control 16 Treatment 36	NA	7 d	mNSS↓
Lee et al. [90]	Intravenous intracerebral	5 × 10^6^ (IV) 1 × 10^6^ (IC) NSCs	Rat Col VII	Sham 30 Control 30 Treatment 120	2 h or 24 h	24 h	MLPT scores↓

Reference	Drug delivery route	Number of cells/exosomes	Type of ICH Model Rat	Sample Size	Treatment day after ICH	Earliest effective time (post ICH)	Behavioral recovery
Sun et al. [60]	Intranasal	1 × 10^6^ MSCs	Mouse Col IV	NA	3 d and 7 d	14 d	mNSS↓ adhesive removal↑ rotarod tests↑ open field tests↑

ABI, autologous blood injection; AD-MSCs, adipose-derived mesenchymal stem cells; Col, collagenase; ESCs, embryonic stem cells; ICH, intracerebral hemorrhage; iPSCs, induced pluripotent stem cells; MLPT, modified limb-placing test; mNSS, modified NSS; MSCs, mesenchymal stem cells; NA, not available; NSCs, neural stem cells; NSS, neurological severity score; VTB, video-tracking box tests; h, hours; d, days; w, weeks; m, months;↑, increased or improved; ↓, decreased; and → , no statistical significance

Interestingly, BM-MSCs can repair the BBB while repairing nerve injury [57, 58]. Chen et al. [58] reported that after intravenous injection of BM-MSCs, the neuromotor function of ICH mice improved by inhibiting inflammation, reducing cell apoptosis, and ultimately reversing the destruction of the BBB. The repair effect of BM-MSCs may be due to endothelial progenitor cells (EPC), which can repair damaged endothelial cells, form new neurons, and promote the recovery of new blood vessels and endothelial function [64, 91, 92]. Pías et al. reported that ICH can cause an increase in EPCs in vivo. The mechanism by which EPC affects cerebral hemorrhage may involve multiple processes, including re-endothelialization, angiogenesis, paracrine mechanisms, and protection of the BBB [93]. Chemokine ligand 12 (CXCL12), also called stromal derived factor-1, is significantly higher in injured areas and has the ability to activate various stem cells [94]. Li et al. utilized endothelial cell (EC) marker tyrosine kinase receptor-2, von Willebrand factor, and VE-cadherin to detect the degree of EPC differentiation and found that by the C-X-C chemokine receptor type 4 pathway, CXCL12 can stimulate EPC differentiation into EC and promote formation of new blood vessels. Of course, there is a dose-dependent relationship between CXCL12 and EPC [92]. EC is also important for nerve repair after ICH injury. Matta and colleagues found that EC could increase the expression of VEGF receptor 3 in NSCs by secreting VEGF-C and improve the survival rate of NCSs. This finding provides hope for the promotion of endogenous nerve cells to treat ICH in the future [95].

In addition to rodent models, studies using primate models have shown promising results. Feng et al. transfected BM-human mesenchymal stem cells into Macaca fascicularis monkeys and found that not only did the clinical test results significantly improve but also that a series of 18F-FDG PET and neurological function scores showed improvement compared to the control group [74].

##### Human umbilical cord mesenchymal stem cells

hUC-MSCs are pluripotent stem cells obtained using a painless procedure. In 2003, Archers et al. isolated and obtained hUC-MSCs from Wharton's jelly (WJ) [96]. Currently, hUC-MSCs are commonly acquired from the umbilical cord or fetal cord blood [97, 98]. Owing to advantages such as abundance, accessibility, low immunogenicity, strong reproductive ability, multi-lineage differentiation potential, and ease of harvesting, hUC-MSCs have been frequently utilized in ICH research [89, 98–104]. Unlike other stem cells, however, they do not seem able to directly replace defective neurons through differentiation into astrocytes or neurons [105–109]. In a rat model, Nan et al. found that after intravenous administration of hUC-MSCs, neurological functional recovery was significantly improved, although rather surprisingly, very few MSC were found in the brain parenchyma [103]. These results suggest that hUC-MSCs do not need to penetrate the brain parenchyma to induce therapeutic effects. To achieve better results, Zhang et al. combined the application of hUC-MSCs with minimally invasive hematoma removal. They reported that this dual treatment was superior to any single treatment and reduced nerve cell death [77]. Acute hematoma removal may reduce hematoma compression, whereas hUC-MSCs directly or indirectly exert neuroprotective effects with the help of soluble substances [77]. A recently published study that combined umbilical cord-derived MSCs with hyperbaric oxygen to treat ICH in mice reported that combined treatment was significantly better than a single treatment [77]. Although they believe that this is inseparable from the low immunogenicity of hUC-MSCs and the effects of hyperbaric oxygen therapy on improving MSCs levels, the specific molecular mechanism remains to be elucidated [110, 111]. WJ-derived mesenchymal stromal/stem cells (WJ-MSCs) are hUC-MSCs with primitive stemness properties. Fasudil is a Rho kinase (ROCK) inhibitor that inhibits the ROCK pathway, which is involved in neuronal functions, including synapse outgrowth and retraction. The microenvironment of ICH is hypoxic compared with that of normal brain tissue [112]. Lee et al. reported that under hypoxic microenvironmental conditions that mimic ICH, fasudil activates WJ-MSCs, which can significantly promote the transformation of WJ-MSCs to neuron-like cells and accelerate the secretion of GDNF to drive endogenous neurogenesis. In addition, neurological deficits in ICH rats were reversed by transferring these primed cells. Interestingly, primed MSCs mediated by fasudil significantly enhanced the number of double-positive cells (microtubule-associated protein 2 (MAP2) + /HuMit +), although the specific molecular mechanism requires further research [113]. In a similar experiment, Liu et al. found that under hypoxic conditions, miR-326 could target the PTBP1/PI3K signaling pathway to reduce the aging of olfactory mucosa MSCs (OM-MSCs), thereby augmenting the therapeutic efficacy of MSCs in ICH. They believe that the reduction in OM-MSC senescence is closely related to an increase in autophagy in OM-MSCs [114]. Placenta-derived mesenchymal stem cells (PSCs) can also be obtained when hUC-MSCs are acquired. In the acute phase after ICH, transfer of PSCs into mice can decrease intracranial pressure and reduce the size of the hematoma, which may be because PSCs increase the number of tight junction proteins, which are related to vascular integrity [115]. Choi et al. [115] also noted that compared with the PSC group, there was a significantly higher level of coagulation factor VII mRNA expression in the control group, indicating that the increased consumption of coagulation factors was the cause of this phenomenon. Interestingly, although PSCs enhanced the motor performance of ICH mice, no evidence of PSC differentiation into astrocytes or neurons was found in lesions surrounding the hematoma [116].

##### Adipose-derived mesenchymal stem cells

AD-MSCs, a type of mesenchymal stem cell with tri-potential differentiation ability, are easily separated from adipose tissue [102]. These cells are not only rich in content and sources but can also induce targeted differentiation [117]. Compared to other stem cells, AD-MSCs have several advantages, such as large numbers, easy accessibility and isolation, stable reproduction, and minimal damage to donors [40, 118, 119]. Therefore, AD-MSCs have attracted significant attention.

In a mouse model of ICH, human AD-MSCs can be used not only to enhance neural function by inhibiting CD11b + CD45 + subpopulation inflammation [120], but also to improve behavioral testing capabilities by differentiating into endothelial lineages [65]. Through in vivo and in vitro experiments, Chen et al. found that the neurological function of mice with cerebral hemorrhage was significantly improved after treatment with rat AD-MSCs (rAD-MSCs). However, no differentiation into endothelial cells was observed. In contrast, these cells transform into neuron-like and glial-like cells, and the expression of neuronal nuclei or glial fibrillary acidic protein is positive. These two discrepancies may be due to differences in cell culture and composition. Therefore, the loss of neuron-like and glial-like cells, secretion of the neuroprotective substance VEGF, and reduction of apoptosis may induce these beneficial effects [62]. Li et al. reported that although stem cell therapy may offer certain advantages, treatment may be delayed owing to the long cell preparation time. To overcome these limitations, utilizing rAD-MSCs with high expression of CX3CR1 can improve the migration ability of transplanted cells in vitro and in vivo and achieve better prognosis [121].

#### Neural stem cells

In 1992, Reynolds and Weiss obtained a self-renewing and multi-differentiation cell from the striatum of the adult mouse brain called neural stem cells (NSC) [24]. This discovery has changed the long-standing notion that nerves cannot be regenerated. Subsequently, an increasing number of NSCs were confirmed to exist in the subgranular zone of the dentate gyrus and the subventricular zone [122]. NSCs are multipotent cells that can differentiate into astrocytes, oligodendrocytes, and neurons [123]. These characteristics have driven scientific research [124]. When ICH occurs, the brain tissue around the hematoma is ischemic and hypoxic, and the cells change from aerobic phosphorylation to anaerobic glycolysis [125]. Owing to the low energy production of anaerobic glucose metabolism, it is particularly important to use limited energy to maintain cell life. In in vitro studies, Lehane et al. [126] found that carbimazole could maintain adenosine triphosphate content by inhibiting the translation process of energy-consuming proteins under hypoxic conditions to protect neurons from hypoxic injury. This undoubtedly reduces nerve damage caused by secondary hypoxia after hematoma. In addition, transplanted NSCs can improve neurological deficits in rodents, but the survival rate of exogenous nerve cells in the host has still plagued researchers. In in vitro experiments using human NSCs (hNSCs), Santilli et al. found that different oxygen concentrations have an important effect on the proliferation and differentiation of nerve cells. They further concluded that mild hypoxic (2.5–5% oxygen) conditions in vitro could promote the proliferation and differentiation of human neural stem cells, which raises hopes for ICH in vivo research [127]. Wakai et al. reported that reactive oxygen species can induce apoptosis in rNSCs transplanted into ICH mice [79]. However, subsequent experiments found that after rNSCs pretreated for 24 h under 5% hypoxia were implanted into ICH rats, hypoxia preconditioning promoted the proliferation of rNSCs. Hypoxic preconditioning accelerated the functional recovery of ICH mice and increased the expression of VEGF in the perihematoma region. The above results may be because hypoxia-inducible factor-1 alpha (HIF-1*α*) upregulates phosphorylated serine threonine kinase/Akt, enhances NSC resistance to hemoglobin cytotoxicity, and improves the proliferation of NSCs [128, 129]. Additionally, Lee et al. confirmed that genetic modification caused hNSCs to overexpress BDNF, GDNF, Akt1, and VEGF and significantly improved the recovery of neurological function in ICH mice [81–83].

In addition to transplanting exogenous stem cells, promoting the proliferation and differentiation of endogenous NSCs is promising. After repetitive transcranial magnetic stimulation in rats with ICH, NSCs directly differentiate into neurons, inhibit their differentiation into glial-like cells, and restore neural function [130]. In addition, transplantation of hESC-derived NSCs into mice with stroke increased endogenous neurogenesis [131]. Although NSCs have broad prospects in the treatment of cerebral hemorrhage, there are understandable ethical issues with NSCs obtained from fetal brains, which hinder their further clinical application [132, 133].

## Mechanism of stem cell treatment in ICH

**Fig. 2:**
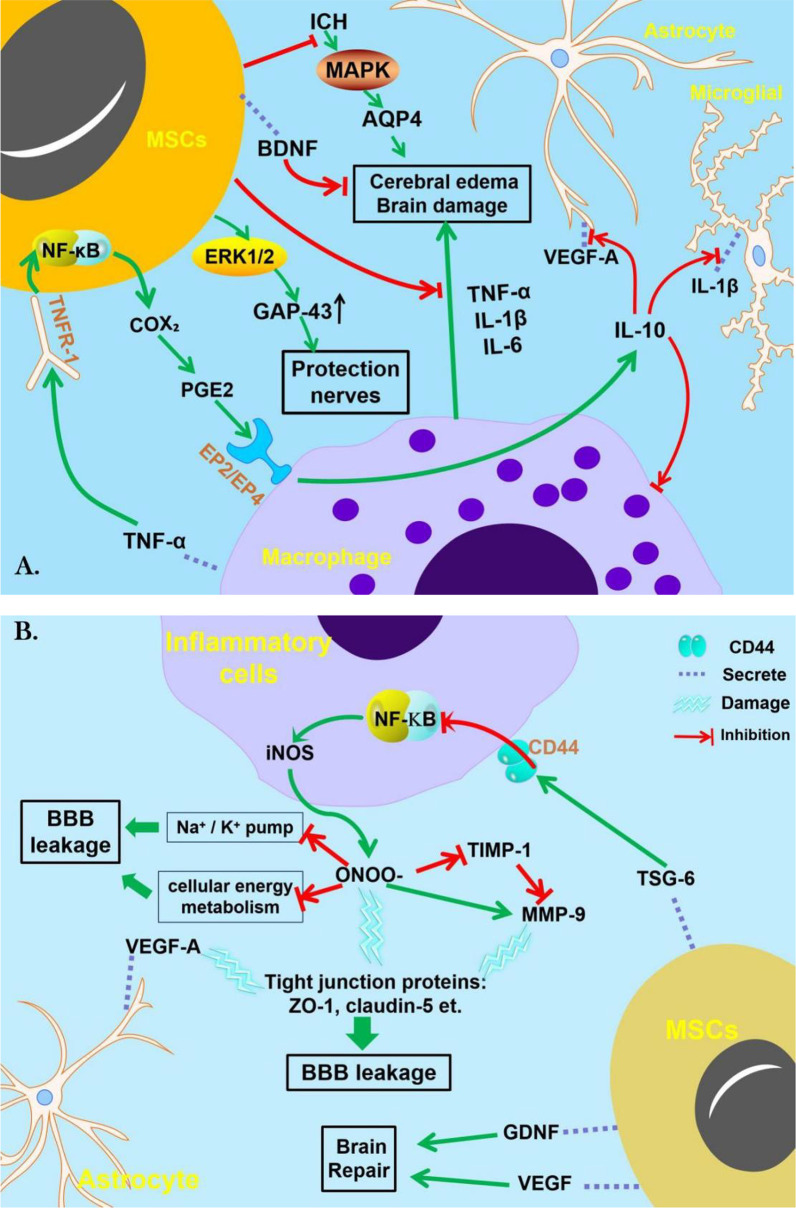
The mechanisms of mesenchymal stem cell therapy for ICH. **A** After ICH occurs, it can cause brain edema through the MAPK signaling pathway. Macrophages can also cause brain tissue edema by secreting cytokines such as TNF-α, IL-1β, and IL-6. In addition to directly inhibiting the above two methods, MSCs can also secrete BDNF to relieve cerebral edema. TNF-α secreted by macrophages can act on MSCs with the help of TNFR-1 and finally increase the level of Prostaglandin E2 (PGE2) through the NF-κB signaling pathway. PGE2 binds to EP2/EP4 on the surface of macrophages to promote IL-10 secretion, thereby inhibiting inflammation. MSCs can directly increase the expression of growth-associated protein-43 (GAP-43) through ERK1/2, thereby exerting neuroprotective effects. **B** Damaged tissue stimulates inflammatory cells (macrophages, astrocytes) to increase peroxynitrite (ONOO–), a strong oxidant, levels through signal pathways. Increased ONOO– can directly damage tight junction proteins and can also promote the production of Matrix metalloproteinase-9 (MMP-9) and damage tight junction proteins, ultimately leading to the blood–brain barrier BBB damage. TIMP-1 as a MMP inhibitor can inhibit MMP activation, but increasing ONOO– can also inhibit the biological effects of TIMP-1. ONOO– can also inhibit sodium potassium pump and cell metabolism to damage BBB. MSCs can secrete TSG-6, which inhibits the NF-κB signaling pathway through CD44, thereby inhibiting subsequent biological processes to improve damaged BBB

Although the mechanism of stem cell treatment in ICH remains unclear, researchers have discovered that it may involve anti-inflammatory mechanisms, neuroprotection, reduction of brain edema, and reversal of BBB damage [58, 60, 63, 78, 82, 134–137] (Fig. 2).

### Relation to inflammation and low immunogenicity

Although hUC-MSCs cannot be directly differentiated into target cells to participate in damage repair, Liao et al. [138] observed that hUC-MSCs can treat ICH by increasing pro-angiogenic activity and suppressing the immune response. The upregulation of a variety of soluble bioactive substances, such as intracellular adhesion molecule-5, BDNF, oncostatin M, macrophage colony-stimulating factor, interleukin-1 receptor antagonist, and ciliary neurotrophic factor, contributes to the immune suppression response [57, 139–143]. Moreover, migrating cells can concurrently weaken leukocyte infiltration and microglial activation [134]. Interestingly, NSCs can not only directly alleviate the functional defects of cerebral hemorrhage by replacing damaged tissue but can also regulate immune cells and some inflammatory factors to play a neuroprotective role. Gao et al. [78] injected NSCs into mouse brain parenchyma and found a neuroprotective effect via modulation of T cell subpopulations and anti-inflammatory (IL-4, IL-10, and transforming growth factor beta), pro-inflammatory [IL-6, interferon gamma (IFN-γ)], and γδ-T cell cytokines. Gao et al. [144] also reported in vivo animal studies and in vitro cellular experiments showing that IL-17 can impede NSC differentiation into astrocytes and neurons and reverse their differentiation by neutralizing anti-IL-17 antibodies. In an ICH mouse model, Chen et al. [110] found that iPSCs-MSC therapy not only improved the recovery of nerve function but also significantly reduced inflammatory factors, including I-κB, NF-κB, TNF-α, IL-β, and inducible nitric oxide synthase (iNOS). iPSCs perform better than BM-MSCs in terms of immunogenic and immunomodulatory properties [145]. Human iPSCs do not express major histocompatibility complex (MHC)-II or costimulatory molecules but only show low levels of MHC-I [42].

### Nerve protection and improvement of neurological symptoms

To study the potential ICH treatment mechanisms, Cui et al. established an accurate ICH model using rat autologous arterial blood. BM-MSCs were injected into rats via the retroorbital vein. At 3 and 7 days after administration, the neurological deficit had improved, and the expression of neurite extension marker growth-associated protein-43 (GAP-43) had increased. When mice were injected with the p-ERK1/2 inhibitor (PD98059) and/or PI3K inhibitor (LY294002), GAP-43 expression was reduced, especially when the two drugs were injected simultaneously. This is the first study to report that PD98059 and LY294002 impede the neuroprotective effect of BM-MSCs. The neuroprotective role of BM-MSC may be achieved by regulating the expression of GAP-43, which can be activated by the ERK1/2 and PI3K/Akt signaling pathways [63]. Cui et al. [146] reported a similar study on a BM-MSC conditioned medium. Chen et al. used IV collagen to establish an ICH mouse model and infused BM-MSCs into the lesions. They found that BM-MSCs can accelerate cell proliferation via the MST1/YAP/Hippo signaling pathway, ensure mesenchymal phenotype switching, and relieve neurological symptoms [147].

### Reduce brain edema

Secondary brain edema after ICH is another cause of brain damage. Zhang et al. confirmed that rAD-MSCs could reduce brain edema, improve motor symptoms, and achieve this goal by limiting inflammation and Aquaporin4 protein expression. Aquaporin4 is an important factor related to cerebral edema, which can be reduced by downregulating the c-Jun N-terminal kinase pathway (JNK) pathway and phosphorylation of p38/MAPK [135].

### Promoting angiogenesis

As a selective interface between the central nervous system and periphery [148], the blood–brain barrier (BBB) is localized at the level of the brain microvascular endothelial cells (BMVECs) that are interconnected together via intercellular junction proteins. Brain pericytes, astrocytes, and neurons communicate with the BMVECs to form the neurovascular unit (NVU). Junction proteins are composed of multiple transmembrane proteins and junctional adhesion molecules. The former includes claudins and occludins, while the latter includes zonula occludens (ZO) and cytoskeleton-related proteins [149, 150]. When ICH occurs, the BBB structure is damaged, which can be improved by EPCs and their secreted external vesicles [64, 91, 92, 151]. Zeng et al. [152] found that EVs secreted by mouse EPCs could promote proliferation, migration, and tube formation of BMECs. In another study on human cells, Loiola Azevedo et al. [151] found that human EPC-secretome decreased the permeability of confluent monolayers of endothelial cells by increasing the expression of occludin, VE-cadherin, and ZO-1. The number of EPCs was positively correlated with the prognosis. This finding has laid the foundation for future cell-free therapies. Annexin A1 (ANXA1), an anti-inflammatory agent, is expressed on microglia, BMVECs, and EVs and plays a role via formyl peptide receptors (FPRs) [153–157]. It has been reported that BBB permeability increases due to endothelial tight junction protein (TJ) degradation and actin microfilament instability when ANXA1 is knocked out [158]. However, ANXA1-FPR2 receptors interact with TJ formation by inactivating the small GTPase, RhoA [158, 159]. Therefore, this is a potential target for future research. In addition, MSCs can promote the maturation of astrocytes and BMVECs, which may be related to the role of VEGF; however, the specific molecular mechanism remains to be studied.

### Protect the integrity of the BBB

The protective effect of BM-MSCs on the BBB can be achieved by increasing the levels of tumor necrosis factor-stimulated gene 6 (TSG-6). Specifically, TNF-α stimulates TSG-6 secretion by BM-MSCs. Increased TSG-6 expression can regulate astrocytes by inhibiting the NF-κB signaling pathway. Elevated TSG-6 levels inhibit iNOS, thereby inhibiting peroxynitrite (ONOO¯) production. Finally, this increase prevents BBB leakage and increases the content of tight proteins such as ZO-1 and occludin, thereby protecting the integrity of the BBB [58]. In addition, BM-MSCs may inhibit NF-κB signaling. When the NF-κB signal is weakened, the activation of MMP-9, which destroys vascular integrity, is hindered [137]. MMP-9 can also be hydrolyzed by endogenous tissue inhibitors of metalloproteinases. It has been shown to improve BBB in rats with stroke by TIMP-1 [160, 161]. Reuter et al. also reported that TIMP-2 gene polymorphism was related to ICH [12]. This may be a potential therapeutic target in the future.

BM-MSCs are very effective in improving endothelial function, avoiding BBB leakage, preventing ICH, and reducing intracerebral hematoma volume by selectively blocking IL-1 and TNF-α [136]. IL-1 and TNF-α gene polymorphisms reportedly increase the probability of rupture and bleeding in cerebral arteriovenous malformations [13, 14]. Future research may bring new hope for the treatment of ICH.

## Exosomes

In the past decade, cell-free therapy has attracted widespread attention. As a safe cell-free therapy, exosomes derived from SCs have been tested in many diseases, such as spinal cord injury, nerve injury, Alzheimer's disease, pancreatic ductal adenocarcinoma, inflammatory bowel disease, and ocular disease [162].

### Source and structure of exosomes

**Fig. 3:**
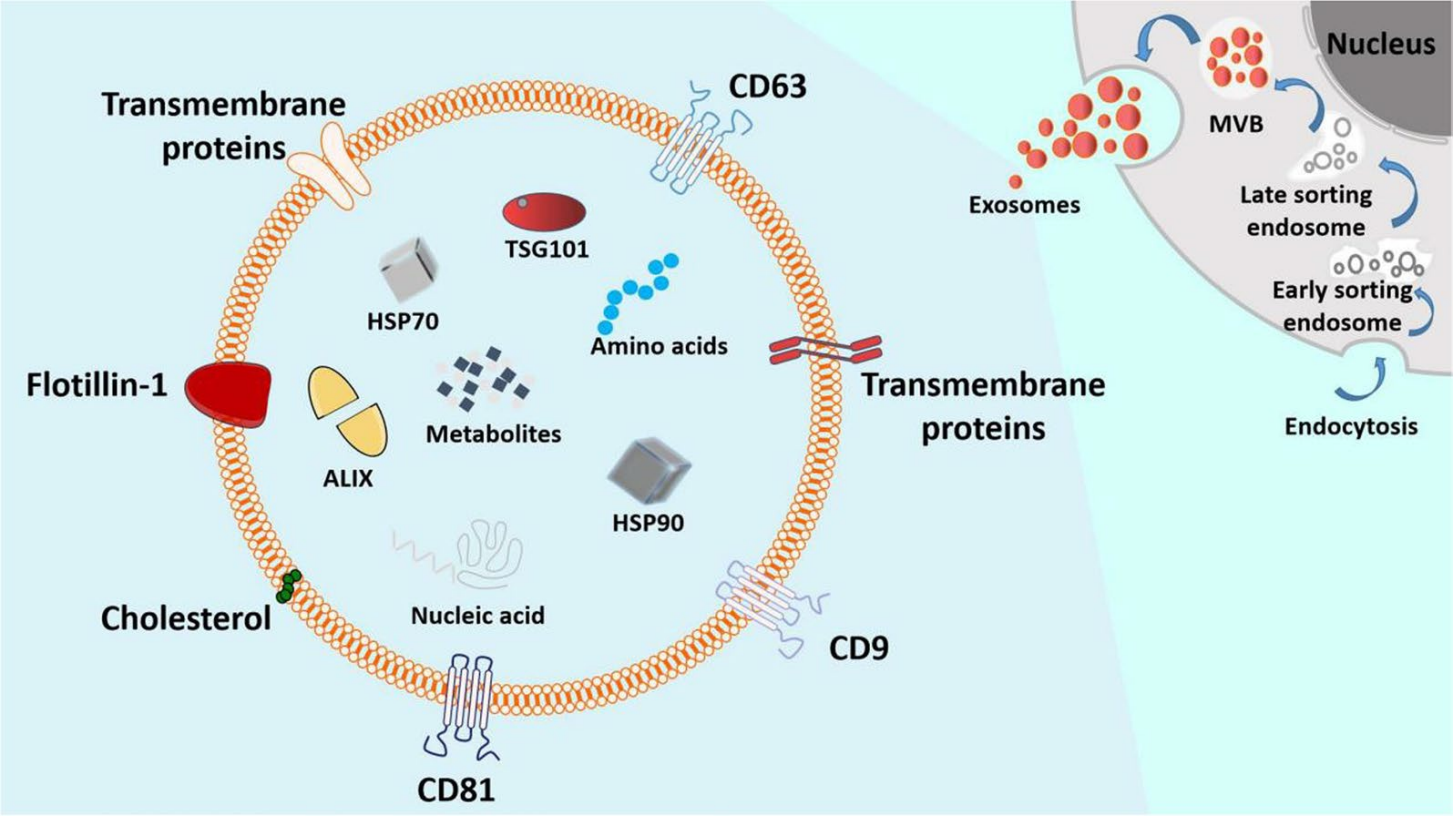
The secretion process and basic structure of exosomes. The cell membrane sags inward to form a multivesicular body (MVB) which then fuses with the cell membrane to discharge exosomes. Exosome-specific marker proteins include CD9, CD81, CD63, flotillin, TSG101, neuroide, and Alix

Heterogeneous vesicles derived from cells are called extracellular vesicles, which are mainly distinguished based on their size, such as exosomes (50–150 nm), microvesicles (100–1000 nm), oncosomes (1000–10,000 nm), and apoptotic bodies (100–5000 nm) [163, 164]. These vesicles may be difficult to distinguish from high-density lipoproteins, low-density lipoproteins, chylous particles, protein aggregates, and cell debris. First, the plasma membrane continuously sags inward to form multivesicular bodies, which then fuse with the plasma membrane. Finally, intraluminal vesicles are released into the extracellular cavity as exosomes [165–167]. Exosomes are microvesicles secreted by stem cells and other cells with a diameter of approximately 40–160 nm and have a lipid bilayer similar to that of a cell membrane. Exosomes from different cell sources contain different components, such as proteins, nucleic acids, lipids, amino acids, and metabolites. Meanwhile, exosomes have marker proteins (CD9, CD81, CD63, flotillin, TSG101, neuroide, and Alix) and some proteins related to exosome biogenesis (Rab GTPases and ESCRT proteins) [163, 164, 166, 168, 169]. Additionally, exosomes also transport RNA, antisense oligonucleotides, and drugs to their intended destinations [167] (Fig. 3). With the increasing attention paid to EVs, an increasing number of molecular components have been found in EVs. Therefore, three different databases have been developed to regroup the molecular data collected during studies on EVs: (i) Vesiclepedia (http://www.microvesicles.org/), (ii) ExoCarta (http://www.exocarta.org/), listing the identified contents of EXOs from multiple organisms, and (iii) EV-TRACK (http://evtrack.org/) using EV-METRIC not only designed as a tool to validate EV studies, but also to generate a wide public EV-TRACK knowledgebase from submitted and previously published experiments [163, 164, 170].

### Exosomes carry substances for the treatment of ICH

Recently, with the in-depth study of exosomes, researchers have begun to experiment with their use in the treatment of ICH. Zhang et al. reported that BM-MSCs can secrete exosomes that highly express microRNA-21. Researchers have found that the therapeutic effect of high-expression microRNA-21 BM-MSCs is to deliver microRNA-21 through exosomes to regulate the transient receptor potential melastatin 7 and the NF-κB pathway, thereby protecting nerve cells [158]. In an autologous arterial blood ICH rat model, Shen et al. injected miR-133b-rich exosomes from BM-MSCs and found that the miR-133b content in the brain tissue increased. This is contrary to the report that ICH in the phosphate-buffered saline (PBS) group reduced the content of miR-133b. Therefore, it is speculated that the improved motor performance by increasing miR-133b after ICH is mediated by targeting blocked RhoA and activating the ERK1/2-CREB pathway [159]. After ICH, the levels of interleukin receptor-associated kinase 1 (IRAK1) and nuclear factor of activated T cells 5 (NFAT5) increased. The former activates the NF-κB signaling pathway to induce a variety of inflammatory responses. The latter can be activated by inflammation and participates in the neuroinflammatory response, promoting the polarization of microglia M1. Duan et al. injected exosomes derived from BM-MSCs loaded with miR-146a-5p into ICH mice via the tail vein. They found that in the BMSCs-miR-146a-5p-exosome group, neuronal apoptosis and inflammation were reduced, microglial polarization was inhibited, and damaged nerve function was significantly improved. They believe that this result is because exosomes transport miR-146a-5p to target cells, which in turn inhibits IRAK1 and NFAT5, thereby reducing ICH damage [171]. Ding et al. used extracellular vesicles derived from BM-MSCs to transfer miR-183-5p into the brains of mice with diabetic ICH. They observed that the inflammatory response was suppressed and behavioral function was improved. They speculated that extracellular vesicles carry miR-183-5p into target cells, and in turn, miR-183-5p targets and reduces programmed cell death 4, thereby inhibiting the expression of nucleotide-binding oligomerization domain-like receptor pyrin domain-containing 3. Ultimately, this inhibits neuroinflammation [172].

### Exosomes are directly involved in the treatment of ICH

MSCs exosomes have a high proliferation rate and exhibit immunosuppressive activity. With increasing maturity levels, the capacity of MSCs to secrete exosomes is reduced [173]. Exosomes may not only serve as carriers for drug delivery [173, 174] but also play a therapeutic role. Otero et al. administered rAD-MSC-derived exosomes into mice via tail vein injection and found recovery of motor function, elevated levels of oligodendrocyte-associated markers, restoration of nerve fiber integrity, and promotion of axonal sprouting [175]. Han et al. injected bone marrow-derived exosomes into ICH mice and discovered that the number of new blood vessels and mature neurons around the hematoma increased and the neurological function of ICH mice improved. Angiopoietin is postulated to increase the density of blood vessels and promote the formation and growth of synapses in the brain. Additionally, by testing the three markers, doublecortin, MAP2, and *β*-tubulin-III, it was found that compared with the PBS control group, the content of subventricular zone precursor cells, which are important for the recovery of nerve function, increased in the exosome-treated group. It was also proposed that white matter increased in the exosome treatment group by increasing the remyelination of newborn microglia [176]. In addition, hUC-MSC-derived exosomes can potentially inhibit apoptosis and accelerate cell reproduction [177]. However, studies regarding hUC-MSC-derived exosomes are only experimental at the level of small animal studies [178], lacking large animal and clinical studies (Table 3).

**Table 3:** Stem cells-derived exosomes application in ICH rats

Source	Type of ICH	Contents	Involved pathway	Main results
AD-MSCs [133]	Col IV	–	–	Improved neurological function, the integrity of fiber tracts, axonal sprout and lesion size
MiR-21-BM-MSCs [126]	Col VII	MiR-21-exosomes	NF-κB	MiR-21 improved the survival rate of MSCs; Exosomes transportation miR-21 reduced neuronal apoptosis; Promoted the recovery of nervous function
MiR-133b-BM-MSCs [134]	ABI	MiR-133b-exosomes	ERK1/2/CREB	Exerted neuroprotective effect; Reduced the apoptosis of nerve cells and neurodegeneration
MiR-146a-5p -BM-MSCs [135]	Col IV	MiR-146a-5p-exosomes	NF-κB	Improved neurological function; Reduced the apoptosis of nerve cells and neurodegeneration; Inhibited inflammation and the M1 polarization of microglia after ICH
BM-MSCs [136]	ABI	Exosomes	–	Enhanced functional recovery; Facilitated endogenous neurogenesis and angiogenesis

*AD-MSCs* adipose-derived mesenchymal stem cells, *BM-MSCs* bone narrow mesenchymal stem cells, *Col* collagenase, *ABI* autologous blood injection, *ICH* intracerebral hemorrhage

## Clinical aspects

With the progress in stroke and stem cell research, many clinical breakthroughs have been achieved. Some clinical and preclinical studies have demonstrated that BM-MSCs may eventually provide a novel, safe, reliable, and effective therapy for ICH, which can enhance nerve function and promote nerve recovery [179–182]. According to a case report published in 2016, researchers injected autologous BMSCs into the ventricles of two patients who were in a minimally conscious state after cerebral hemorrhage. After 12 months, the National Institutes of Health Stroke Scale scores of both patients had improved [183]. In a double-blind, phase I/II randomized controlled trial, autologous BM-MSCs were injected intravenously into patients with ICH. Patients were randomized to receive two intravenous injections of autologous MSCs or placebo 4 weeks apart. Stem cell treatment was concluded to be safe with improved neurological function compared with that of a placebo group [184]. In addition to hemorrhagic stroke, bone marrow mesenchymal stem cells play a pivotal role in clinical studies on ischemic stroke [185–187].

Recently, clinical trials of NSCs for the treatment of non-traumatic cerebral hemorrhage have also achieved exciting results, including transplantation of NSCs into the subarachnoid space of patients with ICH via lumbar puncture, demonstrating that exogenous cells repaired function deficiencies and reduced ICH volume [188]. A clinical follow-up study also confirmed that EPC levels in patients with cerebral hemorrhage are related to prognosis. Seven days after ICH, circulating EPC levels were positively related to prognosis and negatively related to the residual amount of hematoma [189]. From a clinical perspective, this finding may also reveal an appropriate time window for EPC administration, namely within 1 week after cerebral hemorrhage.

Although these results provide a theoretical basis for future applications, the clinical application of BM-MSCs is still progressing. Extracting bone marrow mesenchymal stem cells may be difficult for patients. With age, the normal bone marrow cavity is inevitably replaced by fat cells, making it more difficult to obtain BM-MSCs [190]. Furthermore, with increasing age, the proliferation and differentiation abilities of hBM-MSCs also decrease [191]. On the other hand, and more importantly, these transplanted cells may eventually develop into malignant tumors [192]. Zhu et al. [193] proposed that hMSCs secrete soluble substances, including VFGF, which are of great significance in promoting tumor growth.

## Conclusion and future perspectives

Stem cell therapy has become an attractive potential treatment option for ICH. Stem cells may improve neurological deficits, enhance motor performance, and protect neural cells from damage. Nevertheless, research and application of stem cell therapy for ICH remain immature. At present, the mechanisms that regulate the therapeutic action between the host and migrated cells remain unclear and may involve filling and replacing damaged tissues, accelerating tissue regeneration, improving inflammation, regulating immune responses, and secreting soluble factors and exosomes. In vivo, only hUC-MSCs failed to differentiate into the target cell, but they seemed to be able to indirectly exert therapeutic effects through biologically active substances. At the molecular level, the underlying mechanisms are unclear. Currently, we believe that there are many deficiencies in the research on stem cell therapy for ICH. First, neither of the two animal models completely reproduced the actual pathological process of cerebral hemorrhage. Therefore, an appropriate modeling method should be selected according to the purpose of the study, and better modeling methods are warranted to address this issue. Second, the model animals were mostly selected from healthy adult animals, and there were deviations from ICH patients with other diseases. Third, after ICH, the extracellular microenvironment may impact the transplanted cells, and simple in vitro experiments may not fully simulate the real situation in vivo. More clinical trials should be conducted under suitable conditions. Moreover, although various administration methods have been utilized, the optimal route of administration remains to be determined. Furthermore, issues such as safety, differentiation uncertainty, immune rejection, low number of stem cells reaching the lesion, and tumorigenicity should be considered. Despite these uncertainties, stem cell and exosome therapy remains a promising but challenging treatment option for ICH.

## Data Availability

Not applicable.

## Abbreviations

ABI: Autologous blood injection
AD-MSCs: Adipose-derived mesenchymal stem cells
ANXA1: Annexin A1
apoH: Apoproteins H
ATRA: All-trans retinoic acid
BBB: Blood–brain barrier
BDNF: Brain-derived neurotrophic factor
BM-MSCs: Bone marrow mesenchymal stem cells
BMVECs: Brain microvascular endothelial cells
Col: Collagenase
CXCL12: Chemokine ligand 12
EC: Endothelial cell
EPC: Endothelial progenitor cell
ESCs: Embryonic stem cells
GAP-43: Growth-associated protein-43
GDNF: Glial cell-derived neurotrophic factor
hAD-MSCs: Human AD-MSCs
hESCs: Human ESCs
HIF-1α: Hypoxia-inducible factor-1 alpha
hUC-MSCs: Human umbilical cord mesenchymal stem cells
ICH: Intracerebral hemorrhage
ICI: Intracranial injection
IFN-γ: Interferon gamma
IL: Interleukin
IND: Intranasal deliver
iNOS: Inducible nitric oxide synthase
iPSCs: Induced pluripotent stem cells
IRAK1: Interleukin receptor-associated kinase 1
JNK: C-Jun N-terminal kinase pathway
MAP2: Microtubule-associated protein 2
MHC: Major histocompatibility complex
MMP-9: Matrix metalloproteinase 9
MSCs: Mesenchymal stem cells
MVB: Multivesicular bodies
NESCs: Neuroepithelial-like stem cells
NFAT5: Nuclear factor of activated T cells 5
NSCs: Neural stem cells
NVU: Neurovascular unit
OM-MSCs: Olfactory mucosa MSCs
ONOO¯: Peroxynitrite
PBS: Phosphate-buffered saline
PGE2: Prostaglandin E2
PSCs: Placenta-derived mesenchymal stem cells
rAD-MSCs: Rat AD-MSCs
RhoA: Ras homolog family member A
rNSCs: Rat NSCs
ROCK: Rho kinase
SCs: Stem cells
TJ: Tight junction protein
TNF-α: Tumor necrosis factor-*α*
TSG-6: TNF-*α*-stimulated gene/protein 6
TVI: Tail vein injection
VEGF: Vascular endothelial growth factor
WJ: Wharton’s jelly
WJ-MSCs: Wharton’s jelly-derived MSCs
ZO: Zonula occludens
